# Pulmonary Tuberculosis in Severely-malnourished or HIV-infected Children with Pneumonia: A Review

**DOI:** 10.3329/jhpn.v31i3.16516

**Published:** 2013-09

**Authors:** Mohammod Jobayer Chisti, Tahmeed Ahmed, Mark A.C. Pietroni, Abu S.G. Faruque, Hasan Ashraf, Pradip K. Bardhan, Md. Iqbal Hossain, Sumon Kumar Das, Mohammed Abdus Salam

**Affiliations:** ^1^Clinical Services, icddr,b, GPO Box 128, Dhaka 1000, Bangladesh; ^2^Centre for Nutrition & Food Security, icddr,b, GPO Box 128, Dhaka 1000, Bangladesh; ^3^Research Administration, icddr,b, GPO Box 128, Dhaka 1000, Bangladesh

**Keywords:** Acute pneumonia, Children, HIV, Severe malnutrition, Tuberculosis

## Abstract

Presentation of pulmonary tuberculosis (PTB) as acute pneumonia in severely-malnourished and HIV-positive children has received very little attention, although this is very important in the management of pneumonia in children living in communities where TB is highly endemic. Our aim was to identify confirmed TB in children with acute pneumonia and HIV infection and/or severe acute malnutrition (SAM) (weight-for-length/height or weight-for-age z score <-3 of the WHO median, or presence of nutritional oedema). We conducted a literature search, using PubMed and Web of Science in April 2013 for the period from January 1974 through April 2013. We included only those studies that reported confirmed TB identified by acid fast bacilli (AFB) through smear microscopy, or by culture-positive specimens from children with acute pneumonia and SAM and/or HIV infection. The specimens were collected either from induced sputum (IS), or gastric lavage (GL), or broncho-alveolar lavage (BAL), or percutaneous lung aspirates (LA). Pneumonia was defined as the radiological evidence of lobar or patchy consolidation and/or clinical evidence of severe/very severe pneumonia according to the WHO criteria of acute respiratory infection. A total of 17 studies met our search criteria but 6 were relevant for our review. Eleven studies were excluded as those did not assess the HIV status of the children or specify the nutritional status of the children with acute pneumonia and TB. We identified only 747 under-five children from the six relevant studies that determined a tubercular aetiology of acute pneumonia in children with SAM and/or positive HIV status. Three studies were reported from South Africa and one each from the Gambia, Ethiopia, and Thailand where 610, 90, 35, and 12 children were enrolled and 64 (10%), 23 (26%), 5 (14%), and 1 (8%) children were identified with active TB respectively, with a total of 93 (12%) children with active TB. Among 610 HIV-infected children in three studies from South Africa and 137 SAM children from other studies, 64 (10%) and 29 (21%) isolates of *M. tuberculosis* were identified respectively. Children from South Africa were infected with HIV without specification of their nutritional status whereas children from other countries had SAM but without indication of their HIV status. Our review of the existing data suggests that pulmonary tuberculosis may be more common than it is generally suspected in children with acute pneumonia and SAM, or HIV infection. Because of the scarcity of data, there is an urgent need to investigate PTB as one of the potential aetiologies of acute pneumonia in these children in a carefully-conducted larger study, especially outside Africa.

## INTRODUCTION

According to the WHO global tuberculosis report 2012, there were an estimated 8.7 million new cases of TB (13% co-infected with HIV) and 1.4 million people died of TB, including 9,90,000 deaths among HIV-negative individuals and 430,000 among people who were HIV-positive in 2011 (1). However, among the overall global estimates, the incidence of active TB in children (defined as those aged <15 years) was estimated at 490,000 cases, and the total number of deaths from TB among HIV-negative cases was estimated at 64,000 in 2011 (1). Data from recently-published articles (2,3) suggest that, with accurate diagnosis and good reporting system, children younger than 15 years are likely to contribute 10-20% of the total disease burden due to TB in endemic areas, with a TB incidence estimated at around 50% of that recorded in adults (2). Mortality from pulmonary TB (PTB) is high among children who present with acute pneumonia and severe acute malnutrition (SAM) (4) with or without HIV infection (5). The co-morbidities from acute pneumonia with SAM or acute pneumonia with HIV are a serious problem among under-five children in developing countries (5−7). The duration of symptoms in PTB presenting as acute pneumonia, with cough, fever, anorexia, and failure to thrive, are often less than two weeks. A recently-published systematic review reported that, in addition to the usual respiratory bacterial aetiology in community-acquired acute pneumonia in severely-malnourished children, there are other important causes that remain mostly unexplored, and one of these causes is TB (8). Yet, presentation of PTB as acute pneumonia in severely-malnourished and HIV-infected children has received very little attention, although this is important in the management of pneumonia in children living in communities where TB is highly endemic (8).

### Epidemiology and stratification of risks

The epidemiology of childhood TB with acute pneumonia, irrespective of nutritional or HIV status, has not been well-addressed in medical literature (8,9). In general, children have paucibacillary disease and reduced strength of coughing and rarely contribute to disease transmission compared to adults (10,11). Children with acute pneumonia and severe malnutrition and/or HIV infection may not be different in these characteristics. The natural history of the disease suggests age as the most important risk factor (12). The risk of disease after primary infection with TB is as high as 50% in infants below the age of one year, 10-20% in children aged 1-2 year(s), 5% in children aged 2-5 years, and only 2% in children aged 5-10 years. The risk increases to 10-20% for children older than 10 years (11,13). As in infants, severe malnutrition and HIV infection are also serious risk factors in children (5,14). All of these risk factors are associated with poor cell-mediated immune responses resulting in severe forms of disease after infection with TB (15-17). Of the infected children who progress to disease, approximately 95% will develop disease within 12 months of infection (18). Thus, on the basis of the high exposure rates of TB in endemic countries, all under-five children with SAM and/or HIV infection with acute pneumonia should be categorized as a high-risk group in terms of developing PTB.

In this review, we aimed to examine the aetiologic role of PTB in children with SAM and/or HIV infection presenting with features of acute pneumonia.

## MATERIALS AND METHODS

We conducted a literature search, limited to the English language, to identify reports focusing on tuberculosis in children with SAM and/or HIV infection with acute pneumonia. Severe malnutrition was defined as weight-for-age or weight-for-height z score <-3 of the median of the National Center for Health Statistics (NCHS) (19). We have also included the children with marasmus, or kwashiorkor, or marasmic-kwashiorkor defined according to the Wellcome classification (20). Acute pneumonia was defined as the radiological evidence of lobar or patchy consolidation (6) and/or clinical evidence of severe/very severe pneumonia according to the WHO criteria of acute respiratory infection (21). The databases searched in April 2013 included: PubMed and Web of Science (January 1974 through April 2013). Table 1 summarizes the search strategies and outcomes. All identified abstracts were reviewed, full articles were retrieved and evaluated for those abstracts that suggested potential relevance to our purpose. We included studies reporting culture-positive TB or acid fast bacilli (AFB) by smear microscopy from samples obtained from spontaneous/induced sputum (IS) or gastric lavage (GL), or broncho-alveolar lavage (BAL), or percutaneous lung aspirates (LA) in under-five children with acute pneumonia and SAM and/or positive HIV status.

## RESULTS

A total of 17 studies met our search criteria. Of these, only 6 published studies (4,22-26) relevant to our review provided a total of 747 children (Table 1). The remaining 11 publications (27-37) were excluded as those evaluated the tubercular aetiology of acute pneumonia either without reporting their HIV status or without specifying their nutritional status. One study (38) was excluded from the review because the children were not enrolled in the study due to acute pneumonia, rather acute pneumonia was reported as a finding among a cohort of culture-confirmed TB.

Table 2 summarizes the review results showing six reports, including three from South Africa, one each from the Gambia, Ethiopia, and Thailand that focused on children younger than 5 years. Studies other than those from South Africa (4,25,26) used mainly LA for the isolation of *M. tuberculosis*. Three studies (22-24) from South Africa used mainly BAL or GL, one of these (23) also used IS for the isolation. The diagnosis of PTB was based on identification of *M. tuberculosis* by culture in all studies, except the study from the Gambia where three of their 5 cases of PTB were diagnosed by the identification of AFB only, one by AFB and culture, and the remaining one by culture of *M. tuberculosis* only. The total number of *M. tuberculosis* isolates in all reports combined was 93 out of 747 (12%). There were 610 HIV-infected children in studies from South Africa and 137 SAM children from other studies; 64 out of 610 (10%) HIV-infected children and 29 out of 137 (21%) SAM children were found to have *M. tuberculosis.* Data on CD4 count of HIV-infected children were not available. Nutritional status of children with *M. tuberculosis* was not specified in South African studies, and HIV status of children in other studies was not mentioned.

## DISCUSSION

An observation of overall 12% cases of TB in 747 children with acute pneumonia with SAM or positive HIV status is a very important finding for clinicians in developing countries where the co-morbidity of acute pneumonia and SAM, or positive HIV status is common. The possibility of TB is not often considered in the differential diagnosis of acute pneumonia and, therefore, work-up for TB is not considered in such cases. Pulmonary TB is common in HIV-infected children, potentially due to their reduction in immunity (5,38), which might help the progression of latent TB infection to active TB (5). With the increased uptake of pneumococcal and *H. influenzae* type b vaccine in developing countries, these pathogens will become relatively less important as causative agents of acute pneumonia (39-41) while *M. tuberculosis* might emerge more frequently as an isolated pathogen in children with acute pneumonia in TB-endemic countries, especially those having SAM and/or positive HIV status. On the other hand, once children become infected with TB, this may progress more rapidly into PTB in them compared to other forms of TB (5,38). However, given the natural history of childhood TB, it is very unlikely that TB will result in severe progressive pathology of lungs with features of acute pneumonia. Moreover, children with TB often fail to gain weight and become symptomatic with features of malnutrition. In our review, children with SAM without the mention of HIV status might also have reduced immune status almost similar to that in HIV, and they have a reduced ability to clear infecting organisms, have reduced mucosal immunity and a favourable micro-environment at the point of organism deposition (42), leading to a higher risk of pulmonary co-infection (43). At this point, due to co-infections in the respiratory system, disease progression might become more severe, and children potentially might present with features of acute pneumonia (5). Although the effects of cell-mediated immunity are different in HIV-infected children compared to SAM children, these two conditions are still the risk factors in developing active TB with potential severe ramification in under-five children, especially in TB-endemic settings (4,36). The role of PTB in children with acute pneumonia having any of the above conditions is still unclear.

**Table 1.** Search strategy used in identifying relevant publications and outcome for this review

Database	Strategy and key words used	Total number of matches	Publications relevant for review	Year of publications
PubMed	(pulmonary OR lung) AND (tuberculosis OR TB) AND (infant OR child *) OR (paediatric OR pediatric) AND (pneumonia OR (acute lower respiratory infection) OR ALRI)	455	6	January1974 to April 2013
Web of Science	Tuberculosis AND Pneumonia AND (infant OR child* OR pediatric OR paediatric)	323	6	January1974 to April 2013

*Implies ‘children’ also; Articles were common in both the searches

**Table 2.** *Mycobacterium tuberculosis* in children with acute pneumonia

Author and year of publication	Country	Age (months)	No. of patients	No. (%) of isolates	Main source of sample
With HIV					
McNally LM *et al*., 2007	South Africa	1-59	87	18 (21)	BAL
Zar HJ *et al.*, 2000	South Africa	< 60	100	10 (10)	IS and GL
Madhi SA *et al*., 2000	South Africa	2-60	423	36 (9)	GL
Without HIV					
Adegbola *et al*., 1994	Gambia	3-58	35	5 (14)	LA and IS
Shimeles D and Lulseged S, 1994	Ethiopia	4-60	90	23 (26)	LA
Morehead *et al*., 1974	Thailand	10-50	12	1 (8)	LA

BAL=Broncho-alveolar lavage;

GL=Gastric lavage;

IS=Induced sputum;

LA=Lung aspirate

The higher (21%) identification of PTB in three of the publications included in this review based on percutaneous lung aspirates done in children with SAM without any adverse events is another important finding in this review (4,25,26). It is not common in countries outside Africa, with higher likelihood of missing TB at the time of diagnosis (29,44). Bacteriological diagnosis of pulmonary TB is very difficult in a resource-poor setting where acute pneumonia and SAM are very common and where there is lack of skilled personnel and adequate facilities to perform lung puncture, although the complication from this procedure is very rare (29,44). Moreover, the gold standard of diagnosis culture confirmed TB−is of limited use in severely-malnourished children due to the paucibacillary nature of the disease and poor bacteriologic yields as cavitation happens rarely in the expansion of the primary focus in malnourished children (45). However, the diagnosis of childhood TB in children in developing countries often employs widely-used scoring systems, such as Modified Kenneth Jones criteria (45) and/or WHO criteria published in 2006 (46), which are based on clinical and radiological findings and TST. They often produce inconsistent diagnosis, especially in children in HIV-endemic areas (47). However, the rate of HIV infection in patients with TB has so far remained below 1% in Bangladesh, China, Indonesia, and Pakistan, although it is more common in severely-malnourished children with TB in sub-Sahara and Africa (48). This indicates the potential value and the need for sensitive bacteriology-based diagnostic approaches, particularly in endemic areas where children frequently present with advanced disease (11). The laboratory in the Medical Research Council (MRC) in the Gambia performs lung puncture with a relatively high yield of organisms (4), and it is presently used in a global multicentre pneumonia aetiology research in child health (PERCH) study (49). In this context, with available facilities for careful monitoring and managing complications effectively by trained personnel in children with acute pneumonia and SAM and/or HIV infection, who do not produce sputum, percutaneous lung aspiration would be the diagnostic test of choice for a better isolation rate of TB. This approach potentially promotes early initiation of antitubercular therapy which ultimately helps reduce morbidity and deaths in such populations.

### Limitations

Our review is limited by the fact that we could identify only six relevant publications covering a relatively-small number of children with a tubercular aetiology of acute pneumonia, along with SAM or HIV infection, which might not be mutually exclusive. We were unable to use other publications on the diagnosis of tuberculosis in children with acute pneumonia because of the lack of information on the specific nutritional status or HIV status of the children. This might have led to an under-estimation of the actual frequency of PTB in SAM and/or HIV-infected children with acute pneumonia. Moreover, lack of information on CD4 count of HIV-infected children is another limitation of this review, which makes it difficult to determine the extent of immune deficiency in the HIV-infected children. However, the combination of two groups of children (children with HIV infection or SAM) in evaluating burden of PTB in our review may also be a limitation of this review.

### Conclusions

Our review suggests that PTB may be a common cause of acute pneumonia in severely-malnourished or HIV-infected under-five children. The identification of high frequency of PTB in severely- malnourished or HIV-infected children presenting with acute pneumonia in the reference studies suggests the need for further investigation of the problem, especially in children outside Africa, to better define the tubercular aetiology of acute pneumonia in such children. The information would certainly play an important role for helping clinicians in making decisions about the diagnosis of PTB in such populations, and policy-makers in amending the management guidelines for acute pneumonia in children with SAM to reduce morbidity and deaths.
